# Two years post affordable medicines facility for malaria program: availability and prices of anti-malarial drugs in central Ghana

**DOI:** 10.1186/s40545-017-0103-0

**Published:** 2017-04-28

**Authors:** Alexander Freeman, Anthony Kwarteng, Lawrence Gyabaa Febir, Seeba Amenga-Etego, Seth Owusu-Agyei, Kwaku Poku Asante

**Affiliations:** 1 Georgetown University, 3700 O St NW, Washington, DC 20057 USA; 2 Kintampo Health Research Centre, Ghana Health Service, P.O. Box 200, Kintampo North Municipality, Brong Ahafo Region Ghana

**Keywords:** Malaria, Artemisinin-based combination therapy, Subsidy, Ghana, Kintampo, AMFm, Global fund

## Abstract

**Background:**

The Affordable Medicines Facility for malaria (AMFm) Program was a subsidy aimed at artemisinin-based combination therapies (ACTs) in order to increase availability, affordability, and market share of ACTs in 8 malaria endemic countries in Africa. The WHO supervised the manufacture of the subsidized products, named them Quality Assured ACTs (QAACT) and printed a Green Leaf Logo on all QAACT packages. Ghana began to receive the subsidized QAACTs in 2010.

**Methods:**

A cross-sectional stock survey was conducted at 63 licensed chemical shops (LCS) and private pharmacies in two districts of the Brong-Ahafo region of Ghana to determine the availability and price of all anti-malarial treatments. Drug outlets were visited over a 3-weeks period in October and November of 2014, about 2 years after the end of AMFm program.

**Results:**

At least one QAACT was available in 88.9% (95% CI 80.9% – 96.8%) of all drug outlets with no difference between urban and rural locations. Non-Assured ACTs (NAACT) were significantly more available in urban drug outlets [75.0% availability (95% CI 59.1% – 90.9%)] than in rural drug outlets [16.1% availability (95% CI 2.4% – 29.9%)]. The top selling product was Artemether Lumefantrine with the Green Leaf Logo, a QAACT. There was a significant difference in the mean price of a QAACT [$1.04 USD (95% CI $0.98 – $1.11)], and the mean price of a NAACT in both the urban and rural areas [$2.46 USD (95% CI $2.11 – $2.81)]. There was no significant difference in the price of any product that was available in urban and rural settings

**Conclusion:**

About 2 years after the AMFm program, subsidized QAACTs in Ghana were widely available and more affordable than NAACTs in the Kintampo North District and Kintampo South Municipality of Ghana. The AMFm program appeared to have mostly succeeded in making QAACTs available and affordable.

## Background

In 2012, there were an estimated 207 million cases and 627,000 deaths due to malaria worldwide. Approximately 90% of these deaths occurred in Sub-Saharan Africa [1]. In Ghana, 100% of the population is at risk for year-round transmission of the deadliest strain of malaria, *Plasmodium falciparum* [2]. The burden of malaria is high in Ghana, with about 323 cases per 1000 children under 5 years old per year [3]. In central Ghana, where the Kintampo North Municipality and the Kintampo South District are located, individuals experience approximately 269 malaria infective bites per person per year. The annual prevalence of malaria is 58% among all ages in the Kintampo District [2].

The National Malaria Control Program of Ghana, in an effort to reach their goal of a 75% reduction in malaria morbidity and mortality by 2015, outlined a strategy that included the primary use of Artemisinin Combination Therapies (ACTs) such as Artesunate Amodiaquine (ASAQ) and Artemether Lumefantrine (AL) for the treatment of malaria [4].

ASAQ and AL are artemisinin-based combination therapies with a high therapeutic efficacy [5]. However, the cost is prohibitively high for many Africans [6]. Other drugs used to control malaria in Ghana includes quinine and Sulphadoxine – Pyrimethamine (SP) which are recommended for management of severe malaria and for Intermittent Preventive Treatment in Pregnancy (IPTp), respectively [7].

Private pharmacies and licensed chemical shops are often the first point of care for many Sub-Saharan Africans, who do not visit a hospital or clinic until after initial self-treatment has failed [8]. In the Kintampo region, awareness of the causes of malaria was high but awareness of the first-line treatment, ASAQ, was low [9].

In Ghana, ACTs are sold over the counter in both licensed chemical shops (LCS) and pharmacies. A pharmacy is manned by a registered pharmacist that serve medications based on prescriptions as well as over the counter drugs such as the anti-malarial treatments described in this study. A LCS is manned by a community members who cannot sell prescribed medications but can sell the anti-malarial treatments that are described in this study over the counter as well. For the remainder of this study, pharmacies and LCS are referred to as drug outlets. It was estimated that in 2009, 91.7% of all ACTs imported into Ghana was by the private sector where public facilities often procured their medication [10]. The supply of ACTs to public facilities from the private sector may have been driven by inadequate government funds to import ACTs through the public sector.

The Global Fund created the Affordable Medicines Facility for malaria (AMFm) Program in 2010 as an experimental financing mechanism in 8 pilot countries, including Ghana, to increase the availability, affordability, market share and use of ACTs. AMFm did not provide the products, nor was it involved in the naming or them. The AMFm program provided the funds via a subsidy to purchase only ACTs that they deemed effective. The products the AMFm program subsidized had a Green Leaf logo on the package and are referred to in this study as a Quality Assured ACT (QAACT). One goal of AMFm was to match the price of a QAACT with the cheaper treatments to push out the use of these less effective options. The first subsidized drugs co-paid by the Global Fund with the Green Leaf logo arrived in Ghana in August 2010 [11].

An independent evaluation of the AMFm program showed a significant impact of the program on the availability, price and market share of the subsidized QAACTs across multiple countries [12, 13]. For instance, the availability of QAACTs in Ghana increased in both urban and rural areas by 52 and 35% respectively [14]; the national average price of ACT decreased from $3.42 to 1.13 per treatment in private facilities [11]; in rural areas, the price of ACTs dropped from $2.74 to 0.94 [14]; the market share of QAACTs increased by 45.3% in private health facilities [14]. QAACTs were found to have been available in remote areas of Ghana at the completion of the AMFm program as well [15].

After the pilot program ended in December 2012, the AMFm program was integrated into normal funding procedures of The Global Fund and Ghana continued to receive subsidized QAACTs from the Global Fund until end of 2013 [16].

This research aimed to determine the state of the private anti-malarial market in a predominantly rural area in Ghana about 2 years after the completion of the AMFm program. Specifically, the aims were to determine the background of the drug outlet attendant decided which medicine to recommend, the availability of all anti-malarial products on the market and the mean price of these anti-malarial products across drug outlets located in the town centers, defined as Urban. and the outskirts of the study area, defined as Rural.

## Methods

### Study area

The cross-sectional study was conducted in the Kintampo North Municipality and Kintampo South District. The study area is situated in the forest-savanna transitional zone of the Brong-Ahafo region of Ghana. It covers an area of 7,162 km^2^ with resident population of approximately 135,000 living in 22,000 homes spread among 97 communities as at 2009 [17]. The study area is predominantly rural, similar to other areas in Ghana as defined by the Kintampo Health Surveillance System [17]. Malaria is the most common cause of illness in the area and over the counter drugs from a drug outlet are one of the first treatment methods febrile illness in Ghana [18]. A majority of the population are peasant farmers, similar to other areas of Ghana [17]. The study area was purposively selected because of the presence of research infrastructure.

The towns of Kintampo, Babato and Jema are the urban areas in the Kintampo North Municipality and Kintampo South District. The drug outlets in these towns were considered urban (*n* = 32) while all other drug outlets were considered in a rural area (*n* = 31).

### Drug outlet survey

The study took place from October 2014 to November 2014. A total of 67 drug outlets were included in the study area. Three were private pharmacies and 64 were operating licensed chemical shops (LCS). Only drug outlets that were open at least 1 day per week, had a permanent location and did not receive public funds to operate were surveyed. Four drug outlets did not meet these criteria as they were permanently closed. A total of 63 drug outlets (60 LCSs and 3 Pharmacies) were included in the analysis.

The locations of the drug outlets were distributed across the entire Kintampo North Municipality and South District.

Anti-malarial treatments were grouped into three categories. Quality Assured ACTs (QAACTs) are ACTs that comply with the Global Fund’s ACT chemical regulations and have a green leaf logo printed on its label. Non-Assured ACTs (NAACTs) are ACTs without the Green Leaf Logo e.g. AL, ASAQ and Dihydroartemisinin Piperaquine (DHAP). Other treatments consisted of Amodiaquine, Artesunate, Quinine and SP.

Trained fieldworkers administered a pretested questionnaire to the drug outlet attendants after they had consented to participate in the study. Field workers observed the physical product before marking it as available on the data collection tool. The methodology used in assessing most popular anti-malarial treatment was modeled on methodology described by Davis et. al [10]. Attendants then named the first, second and third most popular anti-malarial chemical composition and its brand over the previous 3 months. The prices of anti-malarial products were determined by asking the drug outlet attendant and observing their price list if they existed. The data collection team was fluent in both English and Twi, the local language.

### Data management and statistical analysis

All data were double-entered using Microsoft Access then transferred to Stata version 13 and R version 3.3.2 for cleaning, analysis and presentation. All products were classified as Quality Assured or Non Assured (with or without Green Leaf Logo) ACT or Other. The products presented are the equivalent of an adult equivalent treatment dose.

Availability of anti-malarial treatments was expressed as simple proportions. Top selling products were determined on a point-based ranking system. For each location, the top selling product was assigned three points, the second two points and the third one point. The points for each product was summed, ranked and described across urban and rural location. Simple proportions available drugs were determined and the statistical difference within subgroups (urban and rural) for their availability were determined using chi-squared tests with *p*-values preset at the .05 level. The price of anti-malarial products were converted from Ghanaian Cedis to U.S. Dollar using the interbank exchange rate at the start of the collection period, October 27, 2014, at 3.2 Cedis = $1.00 USD. Prices are presented as the mean price of each product from all drug outlets that had the product available. The price of an unavailable product was not collected. The data is presented for all drug outlets, and then disaggregated by drug outlets located in urban and rural areas. Welch’s two sample t-tests was used to test significant differences between the mean price of QAACT and NAACT at *p* < .05 level of significance.

### Ethical consideration

Ethical approval was granted from the Kintampo Health Research Centre institutional Review Committee (Federal Wide Assurance number: FWA00011103) in Kintampo, Ghana. All Licensed Chemical Shop and Pharmacy Attendants were read the informed consent form and signed before enrolling in the study. Data was stored on a password protected computer and paper forms were stored in a secure office.

## Results

### Demographics of drug outlet attendants

The average age of a drug outlet attendant was 41.9 years (range: 18 – 73). There was no difference in the average age of rural and urban attendants. A majority of drug outlet attendants were male and there were more male attendants in urban areas compared with rural areas (Table 1). Urban drug outlet attendants had attained a higher level of education. 81.3% of urban attendants had completed secondary school while approximately half (48.4%) of their rural counterparts had done the same. Urban drug outlets had a higher rate of recording sales in a physical book, with 84.4% of urban drug outlet attendants able to show physical proof of the book. There was no significant difference in the age, years of experience in the industry, rate with professional drug-dispensing certificate, or training in malaria treatment in the past 6 months between rural and urban drug outlet attendants. Of those who had attended a training, a majority of them went to one hosted by the local Pharmacy Council.

**Table 1:** Characteristics of Licensed Chemical Shop and Pharmaceutical Attendants in the Kintampo North Municipality and South District

Variable	Urban Shops	Rural Shops	All Shops
Number of Shops	32	31	63
Average Age (Range)	41.8 (20–73)	42.0 (18–65)	41.9 (18–73)
Experience in years (Range)	12.0 (2–30)	13.0 (1–30)	12.5 (1–30)
Gender %, Male	93.8	71.0	82.5
Type of Drug Outlet %, Licensed Chemical Shop	90.6	100.0	95.2
Sales Record %, Keeps Record	84.4	51.6	68.3
Level of Education %, Above Secondary Education	81.3	48.4	65.1
Professional Drug-Dispensing Qualification %, Has Qualification	43.8	38.7	41.3
Malaria Treatment Trained in Past 6 Months %, Attended Training	84.3	71.0	77.8

### Availability

There was a difference in the availability of QAACTs when ungrouped into its two chemical compositions. Quality Assured Artesunate – Amodiaquine (ASAQ) was significantly more available in urban drug outlets than rural drug outlets. 59.4% of urban drug outlets stocked ASAQ with a Green Leaf logo while only 29.0% of rural drug outlets did the same (Table 2). Quality Assured Artemether – Lumefantrine was more available in rural drug outlets, with 87.1% of rural drug outlets having at least one treatment while 75.0% of urban drug outlets doing the same. This finding was not significantly different between urban and rural drug outlets, but it is suggestive of the popularity of AL compared to ASAQ in rural areas.

**Table 2:** Availability of Anti-Malarial Products in the Kintampo North Municipality and South District

Product	Urban Drug outlets	Rural Drug outlets	All Drug outlets	
	Drug outlets with Product Available (%) *N* = 32	Drug outlets with Product Available (%) *N* = 31	Drug outlets with Product Available (%) *N* = 63	
Any QAACT	84.4	93.6	88.9	
QA AL	75.0	87.1	81.0	
QA ASAQ	59.4	29.0	44.4	*
Any NAACT	75.0	16.1	46.0	*
AL	71.9	12.9	42.9	*
ASAQ	21.9	0.0	11.1	*
DHAP	25.0	3.2	14.3	*
Any ACT	90.6	93.6	92.1	
AL	84.4	90.3	87.3	
ASAQ	68.8	29.0	50.8	*
DHAP	25.0	3.2	14.3	*
Any Other	81.3	80.6	80.6	
Amodiaquine	21.9	51.6	36.5	*
Artesunate	0.0	3.2	1.6	
Quinine	31.3	48.4	39.7	
SP	71.9	64.5	68.3	
Any Anti-Malarial	96.9	93.6	95.2	
Total Number of Products Available	6.19	4.03	5.12	*

*Signifies chi-squared value between urban and rural groups is below *p* < .05 level of significance

Amodiaquine was significantly more available in rural drug outlets than in urban drug outlets. 51.6% of rural drug outlets had at least one dose of Amodiaquine in stock while 21.9% or urban drug outlets did the same.

There was no significant difference in the availability of Quinine or Sulphadoxine – Pyrimethamine between urban and rural drug outlets. 39.7% of all drug outlets stocked Quinine and 68.3% of all drug outlets stocked SP.

The total number of variations available was significantly greater in urban drug outlets than in rural drug outlets. In urban drug outlets, customers had a choice between a mean of 6.19 products while customers at rural drug outlets could choose from an average of 4.03 products. 95.2% of all drug outlets stocked at least one anti-malarial product.

It is important to compare the availability of QAACTs and NAACTs in both urban and rural areas as shown in Table 3. QAACTs were significantly more available in rural drug outlets than NAACTs. 93.6% of rural drug outlets carried at least one QAACT while only 16.1% of drug outlets carried a NAACT. There was no significant difference between the availability of QAACTs and NAACTs in urban drug outlets (Table 3).

**Table 3:** Availability of ACTs in Urban and Rural Settings of the Kintampo North Municipality and South District

Product	Urban Drug outlets		Rural Drug outlets		All Drug outlets	
	Product Available (%)		Product Available (%)		Product Available (%)	
Quality Assured AL	75.0		87.1	*	81.0	*
Non-Assured AL	71.9		12.9		42.9	
Quality Assured ASAQ	59.4	*	29.0	*	44.4	*
Non-Assured ASAQ	21.9		0.0		11.1	
Any QAACT	84.4		93.6	*	88.9	*
Any NAACT^a^	75.0		16.1		46.0	

*Signifies chi-squared value between Quality Assured group and Non-Assured group is below *p* < .05 level of significance

^a^This statistic excludes dihydroartemisinin – piperaquine (DHAP), an ACT that was sold in Ghana but not as a QAACT. For that reason, DHAP is not compared in this table

The availability of QAACTs and NAACTs were different as well. There was a significant difference between the availability of any NAACTs and any QAACTS in rural but not urban drug outlets. AL was the most widely available product, Quality Assured or Non-Assured. There was a large difference in the availability of Quality Assured AL and Non-Assured AL in rural areas. 87.1% of rural drug outlets had QA AL while only 12.9% had NA AL (Table 3). No rural drug outlet had NA ASAQ available for sale.

### Top sellers

Artemether Lumefantrine was the top selling anti-malarial product sold in both urban and rural settings in the study area. The top selling brand of this ACT was a product of Ipca Laboratories, India, a Quality Assured product with the Green Leaf logo. The second most popular product differed between urban and rural drug outlets. Quality Assured ASAQ was the second top selling anti-malarial treatment in urban drug outlets and SP was the second top selling in rural drug outlets. Quinine was the third top selling product in rural drug outlets while it was rarely mentioned by attendants among urban drug outlets.

### Price

The mean price of a QAACT [$1.04 (95% CI $0.98 – $1.11)] was significantly different and more than half the price of a NAACT [$2.46 (95% CI $2.11 – $2.81)] (Table 4). The mean price of a QAACT was slightly but significantly more expensive than the mean price of the other combined products [$0.90 (95% CI $0.78 – $1.01)]. The mean price of Amodiaquine was $1.18 (95% CI $1.04 – $1.32), Quinine was $1.45 (95% CI $1.30 – $1.60) and Sulphadoxine – Pyrimethamine was $0.43 (95% CI $0.34 – $0.52), the lowest mean price of any anti-malaria treatment. One Artesunate product was surveyed, priced at $0.94. There were no significant differences in price between urban and rural drug outlets for any specific anti-malarial product.

**Table 4:** Mean Price of ACTs by Label Type in the Kintampo North Municipality and South District

Product	Urban Drug outlets		Rural Drug outlets		All Drug outlets	
	Price (USD)		Price (USD)		Price (USD)	
Quality Assured AL	1.14	*	1.13	*	1.14	*
Non-Assured AL	2.59		2.87		2.63	
Quality Assured ASAQ	0.84		0.83	n/a	0.84	
Non-Assured ASAQ	0.74		-		0.74	
Quality Assured DHAP	-	n/a	-	n/a	-	n/a
Non-Assured DHAP	2.56		-		2.56	
All QAACT	1.02	*	1.07	*	1.04	*
All NAACT	2.42		2.87		2.46	

*Signifies t-statistic between Quality Assured group and Non-Assured group is below *p* < .05 level of significance

- = Not available in any drug outlet

n/a = Test not applicable due to lack of data

However, there was a significant difference in price between a QAACT and its NAACT counterpart between the urban and rural setting. Quality Assured AL was the only chemical composition that was significantly cheaper than its Non-Assured counterpart (Table 4). This was true in the urban and rural setting. Quality Assured ASAQ, however, was not cheaper than the Non-Assured ASAQ in urban or rural settings.

## Discussion

This research describes the state of the anti-malarial market in Kintampo area of Ghana in 2014, about 2 years after the end of the AMFm program. Tracking the effect of a subsidy such as this allows for a better allocation of resources by determining if the amount co-paid by the Global Fund adequately lowered the price to an acceptable price compared to other products on the market. Donor-funded subsidies, such as the AMFm program from the Global Fund, require proof that there was a measurable impact from their actions.

This study found that the availability of QAACTs was high in the study area (>80%) about 2 years after the end of AMFm program especially in rural areas. The high availability of QAACTs found in this study is similar to that found by Tougher et al. in their evaluation of AMFm in Ghana at the end of AMFm program [11]. In the same evaluation Tougher et al. concluded that AMFm had made a significant impact on the availability of QAACTs especially in the public sector [11]. Coming after the Tougher et al. study, this study therefore provides an assurance of a sustained effect of AMFm and meets the estimated bench mark of success of the program about 2 years after its conclusion [19]. Similar observations have been documented in other parts of Ghana [20, 21].

A QAACT as a top-selling product mentioned by drug outlet attendants in the entire study area signals the success of one of the AMFm program’s goals to encourage the sale of ACTs to treat malaria. However, SP and Quinine, neither being recommended treatments, were both highly sold in rural drug outlets. Customers prefer products they have used successfully in the past and were less expensive. Quinine is the recommended treatments for patients with complicated or severe malaria as well as the treatment of uncomplicated malaria in the first trimester of pregnancy [7]. SP is used for IPT for pregnant women. The popularity of these two products is likely explained by its historical use and lower price of SP [22]. These data of top selling products are important for the National Malaria Control Programs to review to understand the preference of the community members in their choice of anti-malarial products for malaria control. On the other hand, the availability of other treatment methods, such as amodiaquine, SP and Quinine, in the drug outlets of the study area is worrying.

The mean price of QAACTs were significantly cheaper than their Non-Assured counterparts across all drug outlets where they were available. This observation is similar to that observed by Ansah et al. [20] and meets the price bench mark for the success of AMFm as found by Tougher et al. [11]. The cheaper price of the QAACTs may have benefitted the consumer as intended. In a study by Adjei et al. [21] in Ghana, community members found ACTs to be affordable during the implementation of AMFm. In addition, there was no significant price difference between urban and rural settings for any ACT However, non-ACT products were significantly cheaper and available in rural areas compared with that in urban areas of the study. It is likely that there is still demand for non-ACT products by sections of the population who still cannot afford ACTs, especially in rural areas. This suggests that AMFm may have generally had a lesser impact in replacing non-ACT products in compared rural areas compared with urban areas.

### Limitations

This research could not conclude that in the absence of the subsidy from the AMFm program, the availability of NAACTs would remain as low in rural drug outlets. QAACTs could be acting as a substitute good that would be replaced by NAACTs if the subsidy from the Global Fund to decrease the price of QAACTs never existed.

The method to determine the top selling products did not require sales data or confirmation from the sales records of the drug outlets. Only verbal confirmation from the attendant was required, introducing the possibility of a response bias. The attendant may have been inclined to respond that a QAACT is the top selling product because they are aware that it is recommended for malaria treatment.

## Conclusion

During the study period of this research, QAACTs were more available and were cheaper than NAACTs in the Kintampo North District and Kintampo South Municipality. The mean price of a QAACT was uniform across urban and rural areas and was slightly higher than the price of non-ACT treatments available.

## Abbreviations

AMFm: Affordable Medicines Facility - malaria
ACT: Artemisinin-based Combination Therapy
AL: Artemether - Lumefantrine
ASAQ: Artesunate – Amodiaquine
DHAP: Dihyrdoartemisinin - Piperaquine
IPTp: Intermittent Preventative Treatment in Pregnancy
KHRC: Kintampo Health Research Centre
LCS: Licensed Chemical Shops
NAACT: Non-Assured Artemisinin-based Combination Therapy
QAACT: Quality Assured Artemisinin-based Combination Therapy
SP: Sulphadoxine – Pyrimethamine
